# Exploring Leadership, Stress of Conscience, and Person-Centered Care in Nursing Homes

**DOI:** 10.1093/geroni/igaa057.587

**Published:** 2020-12-16

**Authors:** Annica Backman, Karin Sjögren, Hugo Lovheim, Marie Lindkvist, David Edvardsson

**Affiliations:** 1 Umea University, Umea, Sweden; 2 Departement of Nursing, Umea, Sweden; 3 Umeå University, Umea, Sweden; 4 Department of Epidemiology and Global Health, Umea, Sweden; 5 La Trobe University, Melbourne, Victoria, Australia

## Abstract

On a daily basis, many care situations contain difficult issues and challenges for care providers. Stress of conscience, such as feelings of guilt, can be experienced by staff when not fulfilling ethical obligations to the residents. Although leadership has been advocated as a key component for staff work perceptions as well as for person-centred care, the impact of nursing home managers’ leadership on levels of stress of conscience among staff and the extent to which person-centred care (PCC) is provided is yet to be explored. Thus, the aim was to explore the relationship between leadership, stress of conscience and PCC as perceived by staff. The study was based on a cross-sectional national survey of 3084 staff and their managers in 189 nursing homes throughout Sweden. Descriptive statistics and regression modelling were used to explore associations. The preliminary results showed that leadership was negatively associated to stress of conscience and positively associated to PCC. PCC were negatively associated to stress of conscience. Additional findings will be presented. This indicates that nursing home managers’ leadership seem to beneficially impact staff work situation in terms of stress of conscience and person-centred care provision.

